# Impact of Discordant Antibiotics on Outcomes After Percutaneous Cholecystostomy for Acute Cholecystitis: A Retrospective Analysis of 184 PCC Patients

**DOI:** 10.3390/jcm14186589

**Published:** 2025-09-18

**Authors:** Lauren Lahav, Nitzan Goldberg, Tamara Jiryis, Hadasa Cristo, Hagai Soback, Shmuel Avital, Yaron Rudnicki

**Affiliations:** Department of Surgery, Meir Medical Center, Faculty of Medicine, Tel Aviv University, Tel Aviv-Yafo 6997801, Israel; lauren.lahav@gmail.com (L.L.);

**Keywords:** bile cultures, percutaneous cholecystostomy, acute cholecystitis, empiric antibiotic therapy, surgical infection management

## Abstract

**Background:** Antibiotic discordance in patients undergoing percutaneous cholecystostomy (PCC) for acute cholecystitis (AC) remains a debated issue. While empiric therapy aims to cover the most common pathogens, source control via PCC may play a greater role in clinical outcomes. This study evaluates the impact of discordant antibiotic treatment on patient outcomes. **Methods:** This single-center retrospective cohort study analyzed 184 PCC procedures performed for AC between 2018 and 2020. Patient demographics, bile cultures, empirical antibiotic regimens, and clinical outcomes were analyzed, with a focus on the impact of discordant antibiotic coverage. **Results:** Of the 184 PCC patients, 128 (69.5%) had positive bile cultures, with *Escherichia coli* (34%), *Enterococcus* (24%), and *Klebsiella* (14%) being the most common pathogens. Resistant bacteria were identified in 28% of patients. Despite 42% (*n* = 78) receiving discordant antibiotics, there were no significant differences in mortality, complications, or length of hospital stay between the discordant and concordant groups. However, the 90-day readmission rate was significantly higher in the discordant group (64.1% vs. 47.2%, *p* = 0.023). **Conclusions:** Although discordant antibiotic treatment did not impact short-term outcomes, it was associated with a significantly higher rate of readmission. These findings suggest that PCC may be the primary driver of acute management; however, inadequate antimicrobial coverage might influence long-term recurrence.

## 1. Introduction

Cholecystectomy remains the definitive treatment for acute cholecystitis (AC). However, many high-risk patients require alternative approaches such as percutaneous cholecystostomy (PCC) for source control, especially patients with significant comorbidities and increased surgical and anesthetic risk [1,2,3]. There is no single universally accepted criterion to define high-risk surgical patients with AC. However, several commonly used parameters—such as frailty assessment, the American Society of Anesthesiologists Physical Status Classification (ASA-PS), and the Charlson Comorbidity Index (CCI)—are well documented in the literature and recent international guidelines [4].

According to the 2018 Tokyo Guidelines: Percutaneous transhepatic gallbladder drainage should be considered the first alternative to surgical intervention in surgically high-risk patients with AC [3,5]. Other methods of gallbladder drainage, such as endoscopic ultrasound-guided or transpapillary drainage, have been reported as alternatives to PCC. While these approaches may reduce recurrence in selected patients, their impact on recurrent AC remains less well established compared with percutaneous drainage [5]. PCC is a generally safe and effective option for high-risk patients who are not surgical candidates, providing symptom relief, infection control, and improved outcomes, although it carries risks such as tube dislodgment, recurrent AC, delayed subsequent cholecystectomy and negative impacts on quality of life [3].

The initial management of AC includes fasting, analgesia, hydration, and the administration of full-dose broad-spectrum antibiotics (ABX) [6,7]. Patients are stratified into three severity levels, designated as Grade 1 (mild), Grade 2 (moderate), and Grade 3 (severe), based on clinical assessment, laboratory findings, imaging, age, and comorbidities [6]. Urgent PCC is indicated in patients with severe AC (Grade III), according to the Tokyo Guidelines 2018, who fail conservative treatment and are considered high surgical risk (CCI ≥ 6, ASA-PS ≥ 3), particularly in the presence of sepsis or organ dysfunction, as in this case general anesthesia may further increase the risk of complications. These high-risk patients are frequently characterized by advanced age and the presence of underlying medical conditions [1,3,5].

Antibiotic therapy is initiated empirically but may be refined based on bile cultures, particularly in cases where resistance patterns are evolving. However, the extent to which empiric antibiotic discordance affects clinical outcomes post-PCC is unclear [8]. In moderate to severe AC (Grade 2 and 3) blood and bile cultures are indicated [7]. The empirical antibiotic treatment largely depends on local bacterial susceptibility patterns. Recent reports indicate a rise in community-acquired antibiotic-resistant bacteria associated with abdominal infections primarily targeting Gram-negative bacteria, the most prevalent causative agents [9,10]. However, in severe cases, there is an indication to broaden antibiotic coverage to include more aggressive pathogens such as *Pseudomonas* and *Enterococcus* [8,11].

Clinical observations indicate that PCC drainage alone can resolve infection, even in cases of antibiotic-resistant bacteria. This study aims to assess the impact of discordant antibiotic therapy on short- and long-term outcomes in PCC-treated AC patients, with a particular focus on readmission rates and delayed cholecystectomy decisions. The novelty of the present study lies in its evaluation of discordant versus concordant empiric antibiotic therapy in PCC-treated patients, an aspect that has not been systematically addressed in previous research.

## 2. Materials and Methods

A retrospective cohort study encompassed all patients who underwent percutaneous cholecystostomy (PCC) for acute cholecystitis (AC) and had bile cultures obtained, at a single academic center from January 2018 to December 2020. Patients who were suspected of harboring resistant pathogens, including those who had undergone prior invasive biliary procedures or drainage, were diagnosed with hospital-acquired cholecystitis, or had recently undergone prolonged antibiotic treatment (>7 days), were excluded from the study. Collected data included demographic information, including age and comorbidities, with a particular focus on diabetes, as some studies suggest a heightened risk for polymicrobial and anaerobic infections [12]. Additionally, clinical, laboratory, and imaging parameters at admission as well as post-procedural outcomes and complications were recorded.

Bile samples were obtained during PCC insertion, and blood cultures at admission. All cultures were processed in the hospital microbiology laboratory using standard methods for bacterial identification and antimicrobial susceptibility testing. Bile and blood cultures were assessed for bacterial growth, strains, and antimicrobial resistance profiles, and concordance of the empiric antibiotic regimen was determined for each patient. Discordant ABX treatment was defined as a regimen that does not fully cover the type of bacteria grown in bile cultures or bacterial strains resistant to the treatment.

Clinical outcomes were assessed including length of hospital stay (LOS), mortality, readmission, need for additional procedures, alterations in antibiotic treatment (escalation or de-escalation), and rates of completion of cholecystectomy. Complications were graded according to the Clavien–Dindo classification for surgical complications [13]. Patients were categorized into the “Concordant group” if their bile cultures revealed bacteria sensitive to the empirical antibiotic therapy they received, or if no bacterial growth was detected. The “Discordant group” consisted of patients whose bile cultures showed bacterial growth, some or all of which were resistant to or not covered by the empirical antibiotic treatment. On subanalysis of the Discordant group, patients who had their ABX treatment adjusted according to the bile culture were referred to as the Late Concordant group and were compared to the initial Concordant group.

Statistical analysis was performed using SPSS version 25.0. Each independent variable distribution was evaluated for normality using the Kolmogorov–Smirnov test. Normal continuous variables were compared between groups using an independent sample *t*-test, while non-normally distributed ones were assessed via the Mann–Whitney U test. Categorical or dichotomous variables were compared using the chi-square test and, as needed, Fisher’s exact test. Significant outcome variables identified in the initial analysis underwent a multivariate analysis, integrating significant background variables. Logistic regression facilitated this multivariate assessment. A *p*-value below 0.05 was considered significant for all analyses conducted.

## 3. Results

Three hundred fifty-six patients underwent PCC insertion during the study period. 172 patients were excluded from the study for not meeting the inclusion criteria: 47 patients were treated for indications other than acute cholecystitis, 83 patients had previous invasive interventions in the biliary tract (PTD, PCC, or ERCP), 20 patients were diagnosed with hospital-acquired cholecystitis or had prolonged antibiotic therapy and 22 patients did not have bile cultures taken during the drainage procedure.

A total of 184 patients were treated with PCC for community-acquired moderate to severe AC. Initially, all patients received empirical antibiotic treatment. Subsequently, due to inadequate improvement and unsuitability for cholecystectomy, all patients underwent gallbladder drainage with PCC insertion.

Bile cultures were obtained in all cases. A group of 56 patients (30%) had negative bile cultures, and an additional 50 (27%) had positive bile cultures with bacteria matching the ABX treatment given. These 106 patients comprised the “Concordant group”. The “Discordant group” included 78 patients (43%), of which 52 cultures (66% of the discordant group) contained at least one resistant bacterium, and 26 (33.3% of the discordant group) had sensitive bacterial strains that were not covered properly by the empirical regimen.

**Figure 1:**
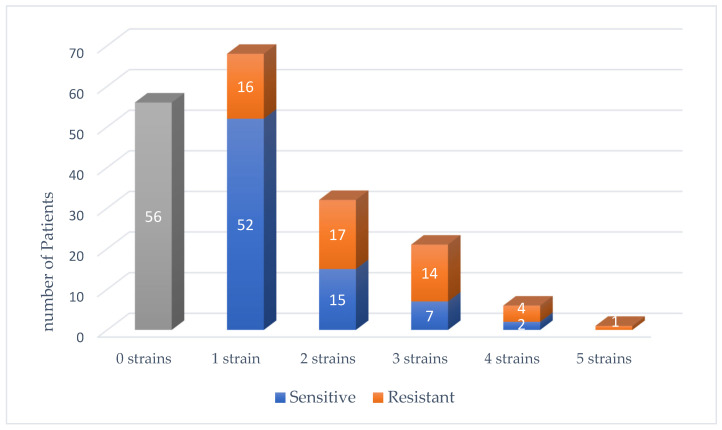
Composition of Bile Cultures in AC: distribution of bacterial Strains and proportion of resistant isolates.

**Figure 2:**
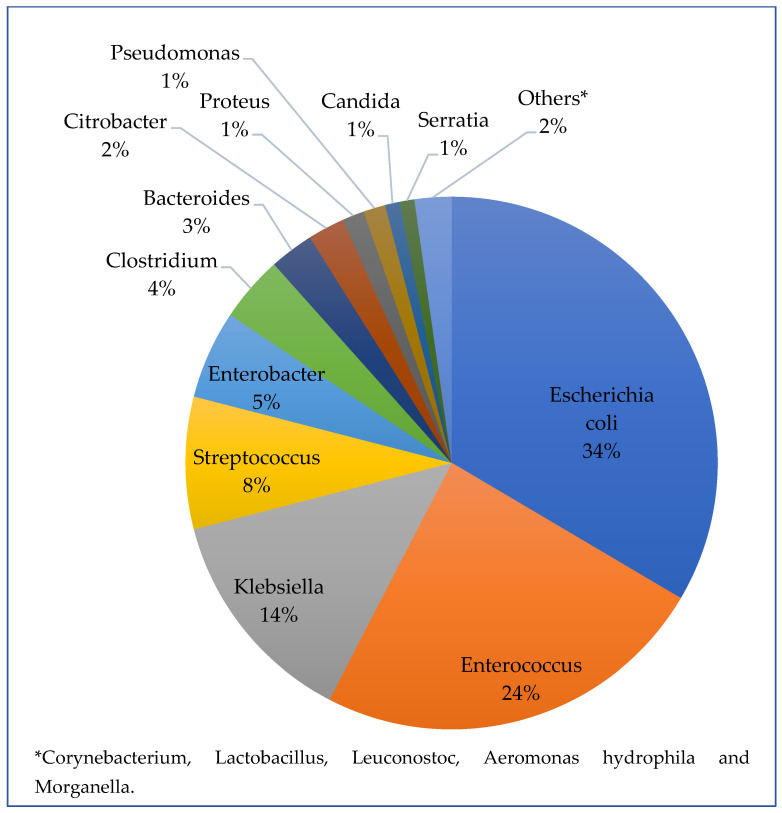
Frequency of bacterial strains isolated from bile cultures in patients with acute cholecystitis.

A review of the number of bacterial strains growing in the bile cultures showed that 56 cultures showed no bacteria at all, 68 had one strain, 32 had two strains, 21 had three strains, 6 had four strains, and 1 patient had five strains of bacteria. Figure 1 shows the composition of bile cultures, including the number of strains found per culture and the proportion of resistant bacteria. Thirty-four percent of cultures had *Escherichia coli* (*E. coli*) bacteria, 24% *Enterococcus*, 14% *Klebsiella*, 8% *Streptococcus*, 5% *Enterobacter*, 4% *Clostridium*, 3% *Bacteroides*, with other species each accounting for less than 2% (Figure 2). Five bacterial strains were isolated in only one culture out of 184 and were grouped as “others”.

When comparing the Concordant group (*n* = 106) to the Discordant group (*n* = 78), aside from the Discordant group being slightly older on average (77.2 ± 12.9 vs. 72.6 ± 13.9 years, *p* = 0.02), the remaining demographic, clinical, and laboratory characteristics were fairly similar, including the distribution of comorbidities, with more than 40% of patients having diabetes mellitus in both groups. More than 75% of patients had right upper quadrant (RUQ) tenderness on physical examination, and approximately one-quarter of patients in each group presented with a fever of 38 °C or above. Both groups had similar sonographic findings such as gallbladder distention and wall thickening. However, the Discordant group exhibited a reduced prevalence of peri-cholecystic findings (21.8% vs. 38.7%, *p* = 0.015). The prevalence of positive blood cultures (bacteremia) was higher in the discordant group but did not reach statistical significance [20 (25.6%) vs. 18 (17%), *p* = 0.3]) (Table 1).

The average duration of the PCC procedure was 49 min, with no significant difference between the groups. The mean time from diagnosis of AC and admission to PCC insertion was shorter in the Discordant group than in the Concordant group (50.5 ± 73.3 vs. 63.7 ± 89.2 h, *p* = 0.045). According to institutional recommendations, Cefuroxime alone or in combination with Metronidazole accounted for over 90% of the empiric antibiotic regimens administered in this study.

After obtaining bile culture results, 20 patients in the Concordant group (18.9%) underwent antibiotic de-escalation due to the growth of susceptible bacteria, while 34 patients (43.6%) in the Discordant group underwent antibiotic escalation due to the growth of bacteria that were resistant to or not appropriately covered by the empiric antibiotic regimen. This implies that 44 patients (56.4% of the Discordant group), persisted with the same narrow-spectrum antibiotic regimen, even though it did not effectively target the bacteria identified in the cultures.

### 3.1. Outcomes and Follow-Up

Following PCC insertion, all patients were followed during hospitalization and post-discharge. The immediate outcome metrics were similar between the two groups. The average length of hospital stay (LOS) was 7.4 ± 6.3 days for the Concordant group vs. 8.6 ± 12.6 days for the Discordant group, *p* = 0.7. The post-procedural complications rate was 48 (45.3%) vs. 42 (53.8%), *p* = 0.25 for the Concordant and Discordant groups, respectively, and mainly bacteremia, bloody discharge in the drain, and early dislodgement of the drain. The rate of Clavien–Dindo complications of grade ≥3 was 13 (12.3%) vs. 13 (16.6%) *p* = 0.4, for the concordant and discordant groups, respectively.

The 90-day readmission rates were notably higher in the Discordant group (64.1% vs. 47.2%, *p* = 0.02), with an average length from discharge to readmission of 28 days for both groups. The leading cause of readmission was acute biliary disease, occurring in 30 patients (59%) in the Discordant group and 25 patients (50%) in the Concordant group. Only 28 patients (33.3%) in the Discordant group and 46 (43.4%) in the Concordant group underwent Cholecystectomy (*p* = 0.2) at an equal average interval of 113 days since PCC insertion. The mortality rate in the Discordant group was 23 (29.5%) vs. 25 (23.6%) in the Concordant group (*p* = 0.36) during a mean follow-up period of 42.75 months for the entire cohort (Table 2). A multivariable analysis was performed, showing that the discordant group retained its elevated risk for readmission even after age adjustment (adjusted OR = 1.96, CI = 1.07–3.6).

### 3.2. Subanalysis of Late Concordant Patients

In a subanalysis of the Discordant group, 34 patients were identified in whom the mismatch between culture results and empiric therapy was addressed by escalating treatment to a broader-spectrum regimen covering the isolated pathogen. This group was named the Late Concordant group and compared to the Concordant group. Other than the group being older (80 ± 11.2 vs. 72.6 ± 13.9, *p* < 0.01) and having a higher rate of patients with fever, most demographics and initial presentations were similar, including gender, comorbidities, specifically DM and HTN, RUQ tenderness, levels inflammatory marker levels, liver function tests, and ultrasound findings. The Late Concordant group had a non-significantly higher rate of post-PCC insertion complications of Clavien–Dindo grade ≥3, as well as a longer hospital stay (LOS) (9.4 ± 8.6 vs. 7.4 ± 6.3 days, *p* = 0.02), and a higher proportion of patients with a LOS of more than seven days (21 (61.8%) vs. 39 (36.8%), *p* = 0.01). They also had a much higher 90 day readmission rate (25 (73.5%) vs. 50 (47.2%), *p* < 0.01), a higher rate of subsequent emergent Cholecystectomy (6 (17.6%) vs. 10 (9.4%), *p* = 0.03), and a higher non-significant mortality rate during the follow-up period (11 (32.4%) vs. 25 (23.6%), *p* = 0.31), (Table 3). A multivariable analysis was performed, showing that the Late Concordant group retained its elevated risk for readmission even after adjusting for age and length of hospital stay (adjusted OR= 3.85, CI = 1.478–10.062, *p* < 0.01).

## 4. Discussion

Optimal empiric antibiotic use requires a delicate balance between ensuring broad pathogen coverage and preventing unnecessary overuse that leads to resistance. Various guidelines point out that before selecting empiric treatment, consideration must be given to local sensitivity profiles, which are unique to each medical institution. Maintaining close surveillance of resistance patterns through local, national, and global antibiograms remains crucial for effective and safe antibiotic strategies [14]. Current guidelines for treating moderate to severe acute cholecystitis recommend broad-spectrum intravenous antibiotic therapy. However, certain medical centers, including the authors’ institution, frequently initiate treatment with a narrow-spectrum empiric antibiotic regimen, targeting prevalent pathogens, pending bacterial culture, and susceptibility assessment from bile samples [12,13]. According to this approach, broad-spectrum antibiotics are administered only when there is reason to suspect a resistant pathogen, such as prior invasive biliary interventions, prior isolation of resistant bacteria, prolonged recent antibiotic therapy, or cases of hospital-acquired cholecystitis. This therapeutic strategy has evolved based on evidence from clinical practice underscoring the central role of source control in managing acute infectious diseases, in this case, through either resection or drainage of the gallbladder [15,16].

The recommended broad-spectrum empirical treatment for moderate-to-severe acute cholecystitis involves potent combinations like Ceftazidime, Vancomycin, and Carbapenems as first-line agents [8,15]. These combinations address bacteria like *Pseudomonas*, *Enterococcus*, and *ESBL*-resistant strains. This study showed that the vast majority, exceeding 90%, of patients diagnosed with moderate to severe AC were managed with a relatively narrow empiric regimen consisting of cefuroxime (second-generation cephalosporin). Given the results of this study and other research in the field, questions arise regarding the justification for the routine use of broad-spectrum antibiotics and their relative contribution to patient treatment compared with source control measures [12,17]. To tackle this significant issue this study aimed to examine the impact of Discordant Antibiotic Treatment on clinical outcomes.

This study aligns with prior literature, demonstrating a bile culture positivity rate of 69.5%. The predominant pathogens were *E. coli*, *Enterococcus*, and *Klebsiella*, consistent with known biliary microbiota trends [18,19,20]. However, the rate of positive blood cultures was somewhat higher than previously reported, at 20.6% compared to the 7.7–15% range found in other studies [8,21]. In this study, *Escherichia coli* was the most common bacterium, found in 34% of bile cultures, consistent with the 31–44% prevalence reported in the literature for biliary infections. Surprisingly, *Enterococcus* was the second-most common bacterium, present in 25% of cultures, despite its highly variable prevalence in the literature (3–34%) [8]. This is particularly notable because the standard empirical antibiotic treatment for acute cholecystitis does not usually cover Gram-positive bacteria. In contrast, anaerobic bacteria (e.g., *Bacteroides*) were found in only 3% of cultures, despite 65% of patients receiving empirical antibiotic coverage for these bacteria. These findings prompt a reevaluation of the routine empiric antibiotic regimen for gallbladder inflammation treatment in our institution.

More than a quarter of bile cultures in the study contained one or more resistant bacteria, a notable percentage considering the patients had no risk factors for resistant pathogens. Additionally, positive cultures from bile and blood are often associated with worse prognoses, including older age and more severe illness, and frequently require percutaneous drainage rather than surgery [20,22,23]. Our study focused on the clinical implications of inadequate empirical antibiotic coverage for these resistant bacteria in the discordant group and its effect on patient outcomes.

Regarding the importance of matching empirical treatment with clinical outcomes and their short- and long-term effects, a 2017 study found that only two-thirds of cases had concordance between the empirical treatment and the bacteria isolated from bile cultures in patients with cholecystitis who underwent percutaneous drainage [17]. Although the study identified poorer clinical outcomes with discordant empirical treatment, it could not demonstrate significant differences due to its small sample size (68 patients).

Existing literature has largely examined bile cultures in cholecystitis but has not definitively addressed the impact of discordant empiric antibiotic therapy on PCC outcomes. Most related studies had small sample sizes (20–150 patients), included mixed diagnoses, or focused on comparisons between percutaneous drainage and cholecystectomy or predictors of positive bile cultures [12,18,19,20,22,23,24,25].

Two recent studies that provide partial insight into this question are worth mentioning. De Miguel-Palacio et al. (2023) examined AC patients, in general, and found that discordant empiric antibiotic therapy was associated with significantly worse outcomes, including nearly a fourfold increase in mortality, prolonged hospital stays, and higher complication rates [26]. Similarly, Wu et al. (2020) evaluated patients with moderate (not severe) AC and reported poorer prognoses in those receiving discordant versus concordant antibiotic coverage [27]. While both studies underscore the clinical importance of appropriate empiric therapy, their limitations in population scope and severity spectrum highlight the need for larger, more targeted investigations.

In our study, the discordance rate for empirical antibiotic treatment was almost two-thirds. A critical question raised by these findings is whether discordant antibiotic therapy contributes to increased readmission due to unresolved infection, or whether bile culture-driven antibiotic adjustments post-discharge could mitigate this risk. While our data suggest that PCC alone provides effective initial infection control, long-term biliary colonization by resistant pathogens may increase susceptibility to recurrence, necessitating further study on prophylactic antibiotic strategies post-PCC.

The older average age in the discordant group may reflect the fact that older age is a risk factor for resistant bacteria in acute biliary infections, as suggested but not definitively proven in the literature [8]. The subanalysis comparing the Late Concordant group with the Concordant group showed that this group, even after adjusting the ABX treatment for the correct bacterial sensitivity, had poorer outcomes, with longer hospital stays, higher readmission rates, and higher mortality, even after adjusting for age and length of stay.

The key limitation of this study stems from its retrospective design and single-center setting, which substantially restrict the generalizability of the findings and underscore the need for validation in larger, prospective multicenter studies. Additional limitations include the absence of data explaining why antibiotic regimens were adjusted in some discordant patients but not in others. Bile culture results with full antibiogram for bacterial susceptibility usually take 72 to 96 h to achieve, and by that time most discordant patients whose antibiotic regimen was not changed had already been discharged due to their clinical improvement after PCC insertion, and thus continued their initial regimen as outpatients. Among those who remained hospitalized when bile culture results became available, clinical decision-making pathways diverged. Patients who showed marked clinical improvement following PCC insertion, despite resistant isolates, were, in consultation with infectious disease specialists, often not escalated to broader therapy, under the rationale that adequate source control had been achieved and escalation was unnecessary, provided that no resistant organisms had grown in blood cultures. By contrast, the Late Concordant group—discordant patients whose antibiotics were adjusted to broader-spectrum ABX—consisted of those who failed to demonstrate adequate clinical improvement following PCC insertion and therefore could not be discharged, a lack of response that may or may not have been attributable to discordant antibiotic therapy. This group also included patients with bacteremia due to resistant organisms, all of whom were escalated to broader-spectrum therapy to ensure adequate coverage of the bloodstream pathogen identified in cultures. Another possible limitation is the lack of data on the proportion of patients with dementia or who were debilitated and bedridden, which are known to be important adverse prognostic factors, particularly in this high surgical-risk population, and may have introduced some degree of bias into the findings.

## 5. Conclusions

Despite a high rate of discordant antibiotic use, short-term clinical outcomes remained comparable between groups, reinforcing the role of PCC in acute management. However, higher readmission rates in the discordant group suggest that antimicrobial coverage may influence long-term recurrence risk. These findings suggest that current empirical antibiotic guidelines for PCC-treated AC patients warrant reevaluation, particularly in light of increasing resistance patterns. Future prospective studies should explore whether targeted therapy based on bile cultures could improve long-term outcomes.

## 6. Recommendations

Our findings suggest that both source control and appropriate antibiotic therapy are key pillars in managing AC, each influencing patient outcomes. We recommend adherence to international guidelines for gallbladder drainage, with rapid risk assessment and early intervention in Grade III patients, where timely source control is critical. At the same time, empiric antibiotics should be tailored to local microbiological patterns through continuous surveillance. Following this study, our institution discontinued routine anaerobic coverage in community-acquired cases due to its low yield, while empiric *Enterococcus* coverage has become more common. Incorporating validated tools such as ASA, CCI, and other risk factors may further help identify patients at risk for resistant pathogens and guide appropriate early broad-spectrum therapy.

## Figures and Tables

**Table 1:** Comparative Analysis of Pre-Intervention and Interventional Variables between the concordant and the discordant groups.

Variables	Concordant *n* = 106	Discordant *n* = 78	*p* Value
Age, (years) mean ± SD	72.64 ± 13.91	77.29 ± 12.91	0.02
Male, *n* (%)	60 (56.6)	45 (57.7)	0.88
Comorbidities, *n* (%)			
Diabetes mellitus	44 (41.5)	33 (42.3)	0.91
Hypertension	67 (63.2)	57 (73.1)	0.15
Dyslipidemia	51 (48.1)	43 (55.1)	0.34
Congestive Heart Failure	16 (15.1)	13 (16.7)	0.77
Ischemic Heart Disease	21 (19.8)	19 (24.4)	0.46
Chronic kidney disease	9 (8.5)	8 (10.3)	0.68
Signs and symptoms, *n* (%)			
RUQ pain	93 (87.7)	64 (82.1)	0.28
RUQ tenderness	84 (79.2)	60 (76.9)	0.70
Fever > 37.9 °C	23 (21.7)	22 (28.2)	0.31
Tachycardia > 99 BPM	25 (23.6)	18 (23.1)	0.93
Laboratory tests, mean ± SD			
WBC count (×10^9^/L)	15.7 ± 6	17.2 ± 7.7	0.25
Bilirubin (mg/dL)	1.4 ± 1.	1.7 ± 2.2	0.52
ALK-P (U/L)	165 ± 1245	169 ± 153	0.56
GGT (U/L)	165 ± 217	169 ± 232	0.52
ALT (U/L)	87 ± 156	86 ± 143	0.45
AST (U/L)	100 ± 186	107 ± 190	0.49
CRP (mg/dL)	18.4 ± 11.6	18.5 ± 12.8	0.98
Ultrasound exam findings—*n* (%)			
GB Distention	98 (92.5)	77 (98.7)	0.08
GB wall thickened	96 (90.6)	76 (97.4)	0.06
GB stones	87 (82.1)	69 (88.5)	0.23
Bile duct enlarged	19 (17.9)	21 (26.9)	0.14
GB infiltration	77 (72.6)	47 (60.3)	0.07
Peri-Cholecystic Findings	41 (38.7)	17 (21.8)	0.01
Positive blood cultures, *n* (%)	18 (17)	20 (25.6)	0.34
PCC Procedure length (min), mean ± SD	45 ± 38	53 ± 97	0.85
Hours from admission to drainage, mean ± SD	63.7 ± 89.2	50.5 ± 73.3	0.04
Empiric Antibiotic regimen, *n* (%)			
Cefuroxime	26 (24.5)	26 (33.3)	0.39
Cefuroxime + Metronidazole	71 (67.0)	50 (64.1)	
Amoxicillin Clavulanate	1 (0.9)	0 (0.0)	
Cefuroxime + Metronidazole + Ampicillin	1 (0.9)	0 (0.0)	
Gentamicin + Metronidazole + Ampicillin	4 (3.8)	2 (2.6)	
Piperacillin + Tazobactam	3 (2.8)	0 (0.0)	

SD = standard deviation, *n* = number, %—percentage, RUQ- right upper quadrant, BPM—beats per minute, WBC—white blood cells, ALK-P—alkaline phosphatase, GGT—Gamma-glutamyl Transferase, ALT—Alanine aminotransferase, AST—aspartate aminotransferase, CRP—C reactive protein, GB—gallbladder, PCC—percutaneous cholecystostomy.

**Table 2:** Comparative Analysis of Post-Interventional Clinical Outcomes Between the Concordant and Discordant Groups.

Clinical Outcomes	Concordant *n* = 106	Discordant *n* = 78	*p* Value
Post-PCC insertion Complications	48 (45.3)	42 (53.8)	0.25
Clavien–Dindo grade ≥ 3	13 (12.3)	13 (16.6)	0.40
Length of hospitalization (days)	7.3 ± 6.3	8.6 ± 12.6	0.68
90 days Readmission rate	50 (47.2)	50 (64.1)	0.02
Interval between admissions (days)	27.7 ± 24.9	28.4 ± 24.1	0.90
Urgent readmission rate, *n* (% of readmissions)	38 (76.0)	41 (82.0)	0.46
Readmission cause, *n* (%)			
Elective for fluoroscopy	9 (18.0)	8 (15.7)	0.75
Acute biliary disease	25 (50)	30 (58.8)	
Difficulty with drain	4 (8.0)	2 (3.9)	
Other Internal Medicine cause	12 (24.0)	11 (21.6)	
Elective Cholecystectomy	36 (34)	17 (21.8)	0.19
Urgent Cholecystectomy	10 (9.4)	9 (11.5)	
Interval from drainage to surgery (days)	113 ± 117	113 ± 124	0.65
Length of follow-up (month), mean	44.2	41.3	0.36
Mortality during follow-up period, *n* (%)	25 (23.6)	23 (29.5)	0.36

Discrete variables are expressed as number and percentage [*n* (%)], and continuous variables as mean ± standard deviation. PCC—percutaneous cholecystostomy.

**Table 3:** Comparative Analysis of Pre-Intervention and Interventional Variables, and Post-Interventional Clinical Outcomes Between the Late Concordant and Concordant Groups.

Variables	Concordant *n* = 106	Late Concordant *n* = 34	*p* Value
Age (years), mean ± SD	72.6 ± 13.9	80 ± 11.2	<0.01
Male, *n* (%)	60 (56.6)	21 (61.8)	0.59
Pre interventional variables			
Diabetes mellitus	44 (41.5)	16 (47.1)	0.57
Hypertension	67 (63.2)	26 (76.5)	0.15
RUQ tenderness	84 (79.2)	26 (76.5)	0.73
Fever > 37.9 °C	23 (21.7)	13 (39.4)	0.04
WBC count (×10^9^/L)	15.7 ± 5.9	18.4 ± 8.1	0.08
CRP (mg/dL)	18.4 ± 11.6	19.4 ± 11.5	0.64
Hours from admission to drainage	63.7 ± 89.2	59.7 ± 106.2	0.04
Post-Interventional Outcomes			
Clavien–Dindo grade ≥ 3	13 (12.3)	8 (23.5)	0.109
Length of hospitalization (days)	7.4 ± 6.3	9.4 ± 8.6	0.02
Patients with LOH > 7 days	39 (36.8)	21 (61.8)	0.01
90 days Readmission rate	50 (47.2)	25 (73.5)	<0.01
Urgent readmission rate—*n* (% of readmission)	38 (76)	22 (88)	0.22
Readmission cause, *n* (%)			
Elective for fluoroscopy	9 (18)	3 (12)	0.78
Acute biliary disease	25 (50)	15 (60)	
Difficulty with drain	4 (8)	1 (4)	
Other Internal Medicine cause	12 (24)	6 (24)	
Urgent Cholecystectomy	10 (9.4)	6 (17.6)	0.03
Mortality during the follow-up period, *n*(%)	25 (23.6)	11 (32.4)	0.31

Discrete variables are expressed as number and percentage [*n* (%)], and continuous variables as mean ± standard deviation (SD). RUQ—right upper quadrant, WBC—white blood cells, CRP—C reactive protein, LOH—length of hospitalization.

## Data Availability

The data presented in this study are available on request from the corresponding author due to privacy and ethical issues.
